# Calcium supplementation and the risk of dementia in the Perth Longitudinal Study of Aging Women: a post-hoc analysis of a randomised clinical trial for fracture prevention

**DOI:** 10.1016/j.lanwpc.2025.101694

**Published:** 2025-10-06

**Authors:** Negar Ghasemifard, Joshua R. Lewis, Simone Radavelli-Bagatini, Simon M. Laws, Blossom C.M. Stephan, Jonathan M. Hodgson, Kun Zhu, Richard L. Prince, Marc Sim

**Affiliations:** a Nutrition and Health Innovation Research Institute, School of Medical and Health Sciences, Edith Cowan University, Joondalup, WA, Australia; b Medical School, University of Western Australia, Crawley, WA, Australia; c Royal Perth Hospital Research Foundation, Perth, WA, Australia; d Centre for Kidney Research, Children's Hospital at Westmead, School of Public Health, Sydney Medical School, The University of Sydney, Sydney, NSW, Australia; e Centre for Precision Health, Edith Cowan University, Joondalup, WA, Australia; f Collaborative Genomics and Translation Group, School of Medical and Health Sciences, Edith Cowan University, Joondalup, WA, Australia; g Curtin Medical School, Curtin University, Bentley, WA, Australia; h Dementia Centre of Excellence, Curtin EnAble Institute, Faculty of Health Sciences, Curtin University, Perth, WA, Australia; i Department of Endocrinology and Diabetes, Sir Charles Gairdner Hospital, Nedlands, WA, Australia

**Keywords:** Healthy aging, Calcium supplementation, Cognitive health, Dementia, Women's health

## Abstract

**Background:**

Concerns have been raised around whether calcium supplements increase dementia risk. This post-hoc analysis of a five-year double-blind, placebo-controlled randomised trial of calcium supplements for primary fracture prevention evaluated the long-term risk for dementia in older women, randomised to either calcium supplements or placebo.

**Methods:**

1460 community-dwelling dementia-free Australian women (≥70 years) were randomised to 1200 mg/day calcium carbonate (n = 730) or placebo (n = 730) for five years and were observed for an additional 9.5 years afterwards. Over 14.5 years, all-cause dementia events (comprising dementia-related hospitalisations and/or deaths) were identified from linked health records. The influence of calcium supplementation on dementia outcomes were examined using Kaplan–Meier survival curves and Cox regression under intention-to-treat (ITT) and per-protocol (PP, ≥80% tablet compliance, n = 830; 50.6% calcium supplements) criteria.

**Findings:**

Mean baseline age was 75.1 ± 2.7 years. Dementia events were recorded in 269 women (18.4%), comprising 243 hospitalisations (16.6%) or 114 deaths (7.8%). No differences in the cumulative dementia-free survival rates were observed between groups in ITT and PP analyses. Compared to placebo, calcium supplements did not increase risk of dementia-related events (unadjusted ITT hazard ratio [HR] 0.90, 95% confidence interval (CI) 0.71–1.15), hospitalisations (HR 0.89, 95% CI 0.69–1.15) or deaths (HR 0.78, 95% CI 0.54–1.13). Similar results were observed in PP analyses.

**Interpretation:**

Calcium supplementation for five years did not increase the risk of all-cause dementia events over 14.5 years in community-dwelling older women. Findings do not support concerns that calcium supplementation increases long-term risk of dementia.

**Funding:**

10.13039/501100000925National Health and Medical Research Council of Australia.

**Research in context**

**Evidence before this study**

We searched PubMed and Google scholar using the terms “calcium supplementation” AND “dementia”/“cognitive function” up until January 2025. Approximately 20% of women over the age of 70 years are affected by osteoporosis with calcium supplementation widely recommended as a preventive measure against bone loss. To date, two observational studies indicate that calcium supplements are associated with greater brain lesion volumes and risk of developing dementia. Alternatively, a post-hoc analysis of a randomised controlled trial reported no relationship between calcium plus vitamin D supplementation and incident dementia. Presently, the long-term effects of calcium monotherapy on dementia risk remains unknown.

**Added value of this study**

Concerns surrounding calcium supplementation and cognitive health may discourage its use for the prevention and management of osteopenia/osteoporosis. This study demonstrates that five years of calcium supplementation (1200 mg per day), as part of a randomised controlled trial to prevent fracture, did not increase the risk of dementia over 14.5 years in 1460 community-dwelling older women.

**Implications of all the available evidence**

These results provide reassurance to patients and clinicians regarding the safety of calcium supplements in the context of cognitive health. This is particularly relevant given the widespread use of calcium supplements in older women to support bone health.

## Introduction

Fragility fractures are a leading cause of disability in older women, a population most at risk of osteoporosis.^1^ Adequate calcium intake is recognised as a fundamental strategy for preventing osteoporosis and reducing fracture risk.^2^ While a well-balanced diet is the preferred means to attain daily calcium requirements, calcium supplements (in conjunction with vitamin D) are often recommended for individuals with osteopenia and osteoporosis.^2^^,^^3^ Some meta-analyses of calcium supplements, with or without vitamin D, have reported an increased risk of adverse vascular outcomes such as myocardial infarction and stroke,^4^^,^^5^ whilst others have found no increased risk.^6^^,^^7^ It has been hypothesised that calcium supplementation may lead to a rapid transient increase in serum calcium levels that may cause intracellular calcium overload, leading to cellular necrosis, or promote the development of calcium deposits in the vasculature, both potentially contributing to the development of dementia.^8^^,^^9^

An observational study of 700 older women (70–92 years old) reported that women taking calcium supplements (n = 98) had approximately 2.99 to 6.77 times higher risk of dementia over five years, specifically in those with a history of cerebrovascular disease.^9^ Similarly, a cross-sectional study of 227 older adults (≥60 years old) reported that calcium supplement users (n = 149) had greater brain lesion volumes, primarily white matter lesions, compared to non-users (n = 78).^8^ To date, there has been no reports from randomised controlled trials (RCT) of calcium monotherapy. However, a post-hoc analysis of an RCT of calcium with vitamin D3 supplements in 4143 older women (65–80 years old) for fracture prevention found no increased risk of mild cognitive impairment or dementia over nearly eight years of follow-up.^10^ It therefore remains uncertain as to whether calcium supplements alone may impact dementia risk.

Given the widespread use of calcium supplements among older women, there is a need to investigate the potential negative impacts of calcium supplements on the risk of dementia in randomised controlled trials of these supplements. We therefore undertook a post-hoc analysis for the long-term risk of dementia from a double-blind, placebo-controlled, five-year randomised trial of calcium supplementation for fracture prevention, in community-dwelling older women.

## Methods

### Participants

Participants were from the Perth Longitudinal Study of Aging Women (PLSAW), originally recruited in 1998 for a five-year RCT examining the efficacy of daily calcium supplementation in preventing fractures, compared to placebo: the Calcium Intake Fracture Outcome Study (CAIFOS).^11^ Ambulant, community-dwelling women aged ≥70 years were recruited from the general population of Western Australia through the electoral roll, a citizenship requirement that covers >98% of this age group. A total of 24,800 potentially eligible women received invitation letters, with 4312 responding and were contacted by telephone. Included participants were dementia-free (Mini-Mental State Examination score ≥25 at baseline), had an expected survival of more than five years, and were not taking bone-active medications, including hormone replacement therapy. Of the 1500 enrolled women, 40 were excluded from this analysis due to calcium and vitamin D supplement prescription. From the remaining 1460 women, 730 were equally allocated, using block randomisation, to the intervention or placebo groups.^11^ Following the five-year intervention (1998–2003), women participated in two subsequent follow-up observational studies (2003–2013), resulting in a total follow-up of 14.5 years. Written informed consent was obtained from all participants. The Human Ethics Committee of the University of Western Australia granted ethics approval (05/06/004/H50, 21st June 1996), and the Western Australian Department of Health Human Research Ethics Committee provided the human ethics approval for the use of linked data (project number #2009/24). CAIFOS and PLSAW studies were retrospectively registered on the Australian New Zealand Clinical Trial Registry (CAIFOS #ACTRN12615000750583 and PLSAW#ACTRN12617000640303) and complied with the Declaration of Helsinki.

### Intervention

Women who were randomised to the calcium group received 600 mg of calcium as carbonate twice daily (1200 mg calcium per day) over a period of five years and were advised to consume the supplements with their morning and evening meals. The placebo group received matched tablets (Wyeth Consumer Healthcare, Baulkham Hills, Australia) following the same regimen. To assess medication adherence, tablets returned at each of the participant's annual check-ups were counted over the five-year period. Adherence was reported as the percentage of the total expected tablet intake. Per-protocol (PP) criteria only included women with ≥80% of tablet adherence for every year across the five-year intervention.^11^

### Dementia outcomes

Dementia outcomes were captured through the Western Australian Data Linkage System over 14.5 years (from 1998 to 2013) and retrieved from the Western Australia Hospital Morbidity Data Collection and mortality records from death certificates, similar to previous publications.^12^^,^^13^ This System captures coded data on all hospital discharges in Western Australia, as well as all death certificate data, including multiple causes of death, in Western Australia. Dementia diagnostic codes were identified using the International Classification of Disease, Injuries and Causes of Death Clinical Modifications (ICD-9-CM) or the International Statistical Classification of Disease and Related Health Problems, 10th version, Australian Modification (ICD-10-AM).^14^^,^^15^ The primary outcome of this study was all-cause dementia events, comprising dementia-related hospitalisations and/or dementia-related deaths. Dementia diagnoses included Alzheimer's disease (ICD-9-CM 290.0, 290.3, 290.10–290.13, 290.20, 290.21, 331.0, ICD-10-AM F00, G30), vascular dementias (ICD-9-CM 290.4, ICD-10-AM F01), and unspecified dementias (ICD-10-AM F03). Dementia events were defined using principal or additional discharge codes within the hospital morbidity data collection. When the coded multiple cause-of-death data was unavailable from linked health records, multiple causes-of-death information from parts one and two of the death certificate (Cause of Death Unit Record File) were used to enhance the identification of dementia cases.^16^ Time-to-event data were calculated as the number of days from the baseline visit until the first dementia-related hospitalisation, loss to follow-up due to death, or the end of the study.

### Covariates

At baseline, clinical and demographic assessments were performed, as previously detailed.^12^ Briefly, assessments included weight (kg) via digital scales and height (cm) via wall-mounted stadiometer, which were used to calculate body mass index (BMI, kg/m^2^). Blood pressure was measured using a mercury column manometer, with the average of three readings. Baseline questionnaires collected data on age, smoking, physical activity, and age at highest level of education. Participants reported their sports and regular physical activities from the previous three months, then activity levels in Kcal/day were calculated using validated methods based on activity type, duration, and body weight.^13^ Smoking status was coded as non-smoker or former/current smoker (defined as smoking >1 cigarette/day for >3 months at any point in time). Participants provided information on their medical history and current medications, which were confirmed with their general practitioner where possible. Additionally, the International Classification of Primary Care-Plus method^17^ was utilised to group various terms representing similar pathological conditions, as classified by the ICD-10 coding system. This approach allowed for the identification of prevalent diabetes (T89001-90009) and atherosclerotic vascular disease (ASVD) based on recorded information. Cardiovascular medications included statins, low-dose aspirin, and antihypertensives. Socio-economic status was determined using the socio-economic indexes for areas developed by the Australian Bureau of Statistics which ranked residential postcodes according to relative socio-economic advantages and disadvantages.^18^ Measurement of total plasma 25-hydroxyvitamin D (25OHD) is described previously.^19^ Baseline dietary calcium intake (mg/day) and alcohol consumption (g/day) were assessed using a validated, self-administered, 74-question semiquantitative food frequency questionnaire from the Cancer Council of Victoria.^20^ This questionnaire was designed to capture habitual dietary intake over the previous 12 months and was completed in small groups under the supervision of a research assistant who provided food models, cups, spoons, and frequency charts to improve accuracy. Apolipoprotein E *(APOE)* genotyping was performed through polymerase chain reaction amplification using oligonucleotide primers, as described previously.^12^

### Focal carotid plaques and carotid intimal medial thickness (CIMT)

Presence of focal carotid plaques and CIMT was evaluated in year three (2001), as described previously.^19^ B-mode carotid ultrasound was used for the assessments, following a standard image acquisition protocol.^21^ To consider the possibility of asymmetrical wall thickening, the arteries were imaged from three different angles: anterolateral, lateral, and posterolateral. The assessments of CIMT and focal plaque were conducted separately on the right and left sides of the carotid arteries. The mean CIMT value was calculated by averaging the measurements from six images (three from each side). Focal plaque was defined as an identified area of focal increased thickness (≥1 mm) of the intima-media layer, and the entire carotid tree was examined for its presence.

### Statistical analyses

Statistical analyses were conducted using IBM SPSS Statistics for Windows (version 29.0, IBM Corporation) and STATA (version 15.1, StataCorp). The primary post-hoc analysis from this study was all-cause dementia-related events, including hospitalisations and/or deaths, with the exposure being the calcium intervention vs. placebo group.^11^ Kaplan–Meier curves and the log-rank tests, as well as Cox proportional hazard modelling were used to evaluate whether women randomised to calcium supplementation had higher risk of dementia outcomes under both intention-to-treat (ITT) and per-protocol (PP) criteria. No violations were detected in the proportional hazard assumption tests for any analysis.

### Additional analyses

**Fig. 1:**
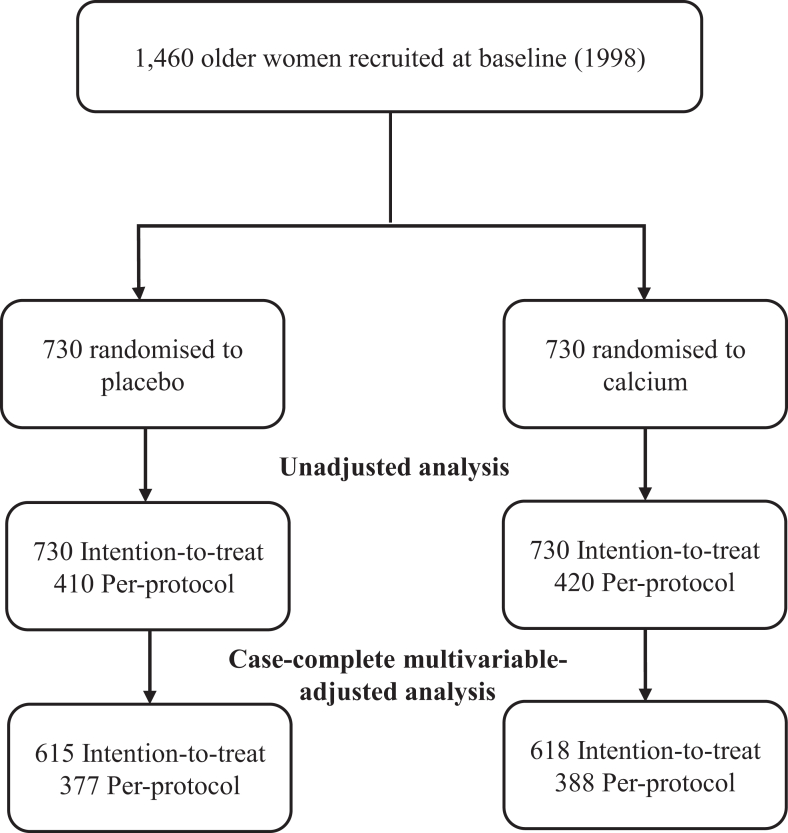
Details of the recruitment, randomisation and follow-up process of participants for the five-year treatment phase of the study. Abbreviations: intention-to-treat (ITT), per-protocol (PP), apolipoprotein E (*APOE*), systolic blood pressure (SBP), body mass index (BMI). Unadjusted ITT (n = 1460) and PP (n = 830) analyses. For case-complete multivariable-adjusted ITT analyses (n = 1233), individuals with missing data (n = 227) comprised of: *APOE* n = 156, SBP n = 37, dietary calcium intake n = 15, socio-economic status n = 11, smoking status n = 6 and BMI n = 2. For case-complete multivariable-adjusted PP analyses (n = 765), individuals with missing data (n = 65) comprised of: *APOE* n = 32, SBP n = 16, dietary calcium intake n = 4, socio-economic status n = 8, and smoking status n = 5.

To account for potential confounders that may influence any relationship between the calcium intervention and dementia outcomes, further multivariable-adjusted Cox regression models were undertaken. For these analyses, 227 women were excluded due to missing covariate data in ITT analyses. This left 615 and 618 women in the placebo and calcium groups, respectively, for the multivariable-adjusted ITT analysis (Fig. 1). We adopted three models of adjustment including: Model 1: unadjusted; Model 2: age, BMI and *APOE* genotype; and Model 3: Model 2 plus systolic blood pressure, use of antihypertensive medication, previous diabetes, prescription of statin medications, use of low dose aspirin, prevalent ASVD, baseline dietary calcium intake, alcohol intake, smoking status, physical activity and socioeconomic status. Although education level is a recognised risk factor for dementia,^22^ this data was not available in our cohort. As a surrogate, we included “age at highest education level” as an additional covariate to Model 3 when considering dementia outcomes. Given the advanced age of the study population, we also conducted competing risks analyses using Fine and Gray's proportional sub-distribution hazards model to account for the competing risk of non-dementia mortality.

As previous work suggested that calcium supplementation might pose a greater risk of dementia in individuals with vascular disease, specifically cerebrovascular disease,^9^ we investigated this potential effect modification by stratifying analyses based on the presence of prevalent ASVD. Due to the small sample size of women with prevalent ASVD (n = 178) compared to those without prevalent ASVD (n = 1282), only age-adjusted Cox regression analyses were conducted in this subgroup. Similarly, due to limited number of women with prevalent cerebrovascular accidents (e.g., ischemic and haemorrhagic stroke, n = 55), Cox proportional hazard models were not adopted for this subgroup. Instead, dementia outcomes over the follow-up period were reported descriptively as proportions based on randomisation groups. Additionally, to account for the potential role of subclinical cardiovascular disease (CVD), the presence of focal carotid plaque and CIMT were added as individual covariates to Model 3 in separate analysis.

To explore whether the incidence rate of dementia outcomes differs based on total daily calcium intake, we classified women into higher and lower intake group based on the median total calcium intake. To account for overall daily calcium intake from both the intervention and/or diet, we first determined the calcium intake from supplementation by multiplying the adherence percentage by 1200 mg/day for participants in the calcium supplementation group only. The calcium intake from supplements was then added to habitual dietary calcium consumption at baseline (mg/day) to estimate total daily intake of calcium. Based on the median total calcium intake of 1610 mg/day as part of PP criteria, women were classified as having higher or lower total calcium intake. Crude incidence rates for dementia outcomes were subsequently calculated in these groups.

Finally, to explore the potential influence of calcium supplementation on cognitive function, we assessed differences in the Abbreviated Mental Test Scores (AMS, as described previously,^23^ n = 1148), at the end of the RCT (year 5, 2003) between the calcium and placebo groups. Given the non-normal distribution of AMS scores, the Mann–Whitney U test was adopted to compare the groups.

### Role of the funding source

The funder of the study had no role in study design, data collection, data analysis, data interpretation, or writing of the report.

## Results

Fig. 1 illustrates the recruitment and categorisation of study participants over the five-year intervention in ITT (n = 1460) and PP (n = 830) analyses. Table 1 presents the baseline characteristics of women who did or did not experience a dementia event over follow-up. This information is also presented by randomisation groups in Supplementary Table S1. Participants mean ± standard deviation (SD) age was 75.1 ± 2.7 years, 37.1% had a history of smoking, 6.5% had a history of diabetes and 12.2% had a history of ASVD. Over a fifth of women (23.3%) carried the ϵ4 allele for the *APOE* genotype. Compared to those who did not develop dementia, participants who progressed to dementia were more likely to be *APOE* ϵ4 carriers (35.3% vs. 20.7%, p < 0.001) and have smoked (45.3% vs. 35.2%, p = 0.002). They were also slightly older at baseline (∼0.8 years, p < 0.001), had lower BMI (∼0.9 kg/m^2^, p = 0.007), and lower tablet adherence (49.1% vs. 58.6%, p = 0.004) (Table 1).

**Table 1:** Baseline characteristics of participants stratified by incidence of dementia.

Baseline characteristics	No dementia	Dementia
Number of participants (%)	1191 (81.6%)	269 (18.4%)
Randomised to calcium supplementation	602 (50.5%)	128 (47.6%)
≥80% tablet adherence (per-protocol), n (%)	698 (58.6%)	132 (49.1%)
Age, years	75.0 ± 2.7	75.8 ± 2.8
Baseline dietary calcium intake,^a^ mg/day	959 ± 354	966 ± 361
Plasma 25OHD,^b^ nmol/L	66.9 ± 29.0	67.6 ± 27.8
Body mass index,^c^ kg/m^2^	27.4 ± 4.8	26.5 ± 4.6
Ever smoked,^d^ n (%)	418 (35.2%)	121 (45.3%)
Systolic blood pressure,^e^ mmHg	137.7 ± 17.6	138.8 ± 20.6
Diabetes, n (%)	71 (6.0%)	24 (8.9%)
Statin medication, yes (%)	226 (19.0%)	50 (18.6%)
Low dose aspirin, yes (%)	242 (20.3%)	66 (24.5%)
Antihypertensive medication, yes (%)	518 (43.5%)	118 (43.9%)
Previous ASVD, n (%)	139 (11.7%)	39 (14.5%)
*APOE* genotypes^f^		
*APOE* ε2/ε3, yes (%)	180 (16.9%)	24 (10.2%)
*APOE* ε2/ε4, yes (%)	25 (2.3%)	4 (1.7%)
*APOE* ε3/ε3, yes (%)	665 (62.4%)	128 (54.5%)
*APOE* ε3/ε4, yes (%)	185 (17.4%)	66 (28.1%)
*APOE* ε4/ε4, yes (%)	11 (1.0%)	13 (5.5%)
Physical activity,^g^ Kcal/day	110 (23–199)	114 (10–220)
Alcohol intake,^h^ g/day	1.9 (0.3–10.0)	1.3 (0.1–8.9)
Socio-economic status,^i^ n (%)		
Top 10% most highly disadvantaged	50 (4.2%)	16 (5.9%)
Highly disadvantaged	146 (12.4%)	29 (10.8%)
Moderate—highly disadvantaged	191 (16.2%)	45 (16.7%)
Low–moderately disadvantaged	184 (15.6%)	40 (14.9%)
Low disadvantaged	253 (21.4%)	51 (19.0%)
Top 10% least disadvantaged	356 (30.2%)	88 (32.7%)

Data are expressed as mean ± SD, median (IQR), or n (%).

Abbreviations: 25OHD, 25-hydroxyvitamin D; ASVD, prevalent atherosclerotic vascular disease; *APOE,* apolipoprotein E genotype; mmHg, millimetres mercury.

a n = 1445.

b n = 1350.

c n = 1458.

d n = 1453.

e n = 1412.

f n = 1301.

g n = 1458.

h n = 1445.

i n = 1449.

**Fig. 2:**
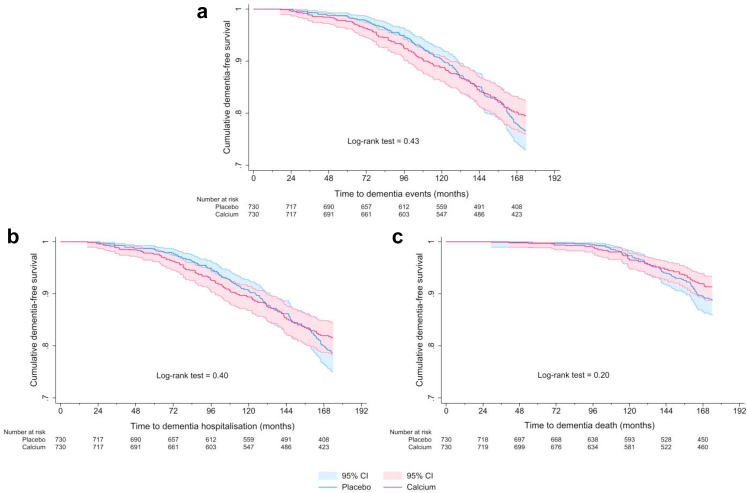
Intention-to-treat Kaplan–Meier survival curves for calcium and placebo groups for a dementia event (a), hospitalisation (b) or death (c) in 1460 women.

**Fig. 3:**
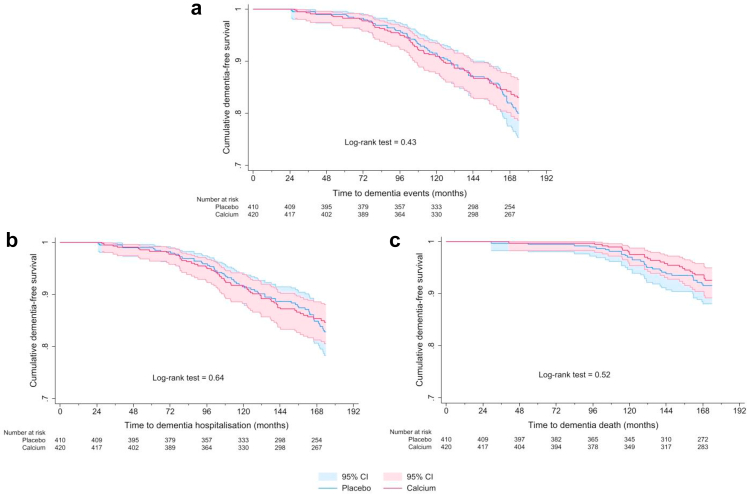
Per-protocol Kaplan–Meier survival curves for calcium and placebo groups for a dementia event (a), hospitalisation (b) or death (c) in 830 women.

Over 14.5 years of follow-up, 269 women (18.4%) experienced a dementia event, 243 (16.6%) had a dementia hospitalisation (mean ± SD follow-up 12.03 ± 3.6, ∼17,570 person-years), and 114 (7.8%) dementia death (mean ± SD follow-up 12.46 ± 3.4, ∼ 18,192 person-year). The mean ± SD age at the time women experienced a dementia event was 85.3 ± 3.9 years, 84.9 ± 3.8 years for dementia hospitalisation, and 87.2 ± 3.6 years for dementia death. ITT and PP Kaplan–Meier survival curves for the placebo and calcium groups over follow-up are presented in Figs. 2 and 3, respectively. Log-rank tests indicated no significant differences (p > 0.05) between the placebo and calcium groups for any dementia outcomes. Unadjusted ITT and PP Cox regression analysis for dementia outcomes are presented in Table 2 and Table 3, respectively, with no statistically significant differences observed between groups.

**Table 2:** Unadjusted intention-to-treat hazard ratios for dementia events, hospitalisations, and deaths over 14.5 years according to randomisation groups.

	Participants (n)	Placebo	Calcium	p value
		730	730	
Dementia events	Events n (%)	141 (19.3)	128 (17.5)	
	HR (95% CI)	Ref.	0.90 (0.71, 1.15)	0.43
Dementia hospitalisations	Events n (%)	128 (17.5)	115 (15.8)	
	HR (95% CI)	Ref.	0.89 (0.69, 1.15)	0.40
Dementia deaths	Events n (%)	64 (8.8)	50 (6.8)	
	HR (95% CI)	Ref.	0.78 (0.54, 1.13)	0.20

Values are hazard ratios (HR) and 95% confidence intervals (CI).

**Table 3:** Unadjusted per-protocol hazard ratios for dementia events, hospitalisations, and deaths over 14.5 years according to randomisation groups.

	Participants (n)	Placebo	Calcium	p value
		410	420	
Dementia events	Events n (%)	70 (17.1)	62 (14.8)	
	HR (95% CI)	Ref.	0.87 (0.61, 1.22)	0.43
Dementia hospitalisations	Events n (%)	60 (14.6)	56 (13.3)	
	HR (95% CI)	Ref.	0.91 (0.63, 1.32)	0.64
Dementia deaths	Events n (%)	29 (7.1)	25 (6.0)	
	HR (95% CI)	Ref.	0.84 (0.49, 1.43)	0.53

Values are hazard ratios (HR) and 95% confidence intervals (CI).

### Additional analyses

In multivariable-adjusted Cox regression models, results remained comparable for both ITT and PP analysis (Supplementary Table S2 and Supplementary Table S3). The addition of age at highest education level to Model 3 did not alter the interpretation of our results (Supplementary Table S4). Competing risks analyses for non-dementia deaths did not substantively alter the results (Supplementary Table S5).

Age-adjusted hazard ratios (HR) and 95% confidence intervals (CI) for the association between calcium supplementation and dementia events over 14.5 years in 178 women with a history of ASVD and 1282 women without ASVD are presented in Supplementary Table S6. No significant differences in the risk for dementia events were recorded between the calcium and the placebo groups. Additionally, when considering only women with prevalent cerebrovascular accidents (n = 55), the proportion of individuals who experienced any dementia events over follow-up was comparable in both the calcium (23.8%, 95% CI 8.2–47.2%) and placebo (26.5%, 95% CI 12.9–44.4%) groups (Supplementary Table S7).

Adding the presence of focal carotid plaque (Supplementary Table S8) or CIMT (Supplementary Table S9) to Model 3, to account for subclinical CVD, did not reveal any differences in the HRs for any of the dementia outcomes between the calcium and placebo groups.

Supplementary Table S10 presents PP unadjusted incidence rates (per 1000 person-year) of dementia events, hospitalisations, and deaths in women, dichotomised by the median of total calcium intake coming from supplements and/or diet. Incidence rates for all dementia outcomes were comparable between groups.

There was no significant difference (p = 0.28) in AMS scores between the calcium (n = 569) and placebo (n = 579) groups at the end of the RCT; median (IQR): 10 (9–10) in both groups.

## Discussion

In this post-hoc analysis of a double-blind, placebo controlled randomised clinical trial, originally designed to assess the effect of five years calcium supplementation (1200 mg/d) on fractures in community-dwelling older women, no evidence for an increase in the long-term risk of all-cause dementia was observed in those randomised to calcium monotherapy. This is supported by both the ITT and PP analyses where calcium supplementation did not increase the risk of dementia events, hospitalisations, or deaths over 14.5 years. When the analysis was adjusted for a range of lifestyle factors (e.g., alcohol intake, socioeconomic status, dietary calcium, smoking, physical activity), prevalent cardiometabolic disease (e.g., diabetes, ASVD), and genetic risk factors (e.g., *APOE* ε4), interpretation of the results remained unchanged.

Similar null findings have been reported in a sub-study of the Women's Health Initiatives as part of a post-hoc analysis of a randomised double-blind placebo-controlled trial.^10^ This study, included 4143 women aged 65–80 years, who were supplemented with 1000 mg of calcium and 400 IU of vitamin D per day for a mean follow-up of 7.8 years.^10^ They reported a non-significant HR for both incident dementia (HR 1.11, 95% CI 0.71–1.74) and mild cognitive impairment (HR 0.95, 95% CI 0.72–1.25) in the supplement group compared to those receiving placebo.^10^ Our post-hoc analysis of a randomised controlled trial of calcium monotherapy supports the findings of calcium with vitamin D supplementation.

Conversely, in 2016, Kern et al.^9^ conducted a five-year longitudinal, population-based study of 700 women (70–92 years) without dementia at baseline and reported higher odds (Odds Ratio [OR] 2.10, 95% CI 1.01–4.37) of developing dementia in the 98 women taking calcium supplements (84 concurrently with vitamin D) compared to the non-users. On close inspection, such findings stemmed from a sub-group analysis where the association between calcium supplementation and incident dementia was confined to women with cerebrovascular disease (those with ischemic white matter lesions or a stroke that occurred prior to baseline or during the follow-up period). Notably, among 108 women in the stroke subgroup, only 15 (13.9%) were using calcium supplements, and six of these individuals developed dementia within five years (OR 6.77, 95% CI 1.36–33.75). Similarly, in a subgroup of 316 women with white matter lesions, 50 (15.8%) were calcium users, of whom 11 dementia cases were recorded (OR 2.99, 95% CI 1.28–6.96 compared to non-users).^9^ This study also did not report the duration and dosage of supplementation. Instead, it categorised participants as calcium users based on drug dispensing list and assumed that they adhered to Swedish recommended daily calcium intake.^9^ Consequently, these women were more likely to be osteoporotic and taking calcium supplements for fracture prevention. This is supported by the incidence of fractures, which was almost double in calcium users (41%) compared to non-users (21%). Considering that fractures are a known risk factor for dementia,^24^ this may have contributed to the results. In our study the proportion of women experiencing a fracture over follow-up was comparable in the calcium (27%) and placebo groups (29%) (analysis not shown). The aforementioned findings^9^ are consistent with a small cross-sectional study by Payne et al.,^8^ where 149 older adults aged ≥60 years, who self-reported calcium supplement use via questionnaire (65% female), had 1.4 times greater brain lesion volume, a risk factor for dementia, compared to 78 non-users, independent of the dosage and duration of calcium supplement used. Both studies were relatively small observational studies that are likely susceptible to unmeasured confounding, including the indication for calcium supplementation such as low bone mineral density or prescription of bone active medications.

Notably, our study is a post-hoc analysis of a five-year RCT with both ITT and PP analyses undertaken. Compared to the study by Kern et al.,^9^ our study design strengthens the validity of our findings. Specifically, calcium supplementation was provided directly to participants, offering more accurate data on dosage and duration of use than reliance on drug dispensing lists. Our study also has a longer follow-up period (14.5 years vs. 5 years) with events identified through two separate datasets: hospital morbidity data collection and mortality records. Consistent null findings for dementia hospitalisations and deaths separately support the overall null results. Additionally, with our larger sample size (n = 1,460, 730 randomised to calcium and 730 to placebo) compared to the previous report (n = 700, only 98 taking calcium supplements),^9^ combined with greater number of dementia events (n = 269 vs. n = 59) over the follow-up period, we were adequately powered to detect any similar associations between calcium supplement use and dementia risk. Unlike previous observational studies,^8^^,^^9^ we also considered tablet compliance. Although compliance rates were similar between the calcium (57.5%) and placebo groups (56.2%), further adjusting the model for this variable showed that higher tablet compliance was associated with a lower relative hazard of dementia events in both the unadjusted (36%) and multivariable-adjusted (27%) analyses (data not shown). This is perhaps unsurprising given that women with lower tablet compliance may have had a greater comorbidity burden contributing to earlier cognitive decline. However, no interaction between compliance and randomisation to calcium vs. placebo was recorded (p > 0.731 for both unadjusted and multivariable-adjusted). Finally, given the advanced age of the cohorts, the use of Cox proportional hazard models that censor for mortality in the current study, would also be preferential to logistic regression^9^ when considering incident dementia.

Calcium supplementation has been hypothesised to increase the risk of dementia through vascular calcification or neuronal damage.^8^^,^^9^ Ancillary studies showed that calcium supplementation did not increase the risk of carotid atherosclerosis and atherosclerotic vascular disease in the current cohort.^19^^,^^25^ Our analysis also revealed no association between randomisation to five years of calcium supplementation and dementia events in women with prevalent ASVD. Additionally, adjusting for structural subclinical CVD (presence of plaque and CIMT) did not affect these findings. Collectively, this suggest that calcium supplementation for five years is unlikely to influence subclinical CVD and dementia risk in older women.

This study has several strengths, including being a large RCT that explores the long–term relationship between calcium monotherapy and dementia outcomes in a cohort of community-dwelling older women, a high-risk population for osteoporosis most likely to be prescribed calcium supplements. Moreover, we considered a broad range of potential risk factors including lifestyle, detailed medication history, cardiovascular risk factors, and *APOE* genotypes. Assessing both habitual dietary calcium intake and calcium tablet adherence is another strength. Further, we undertook both ITT and PP analyses (≥80% adherence to the intervention), reporting similar results.

Despite its strengths, our study has limitations. As this study is comprised of older White women, whether such results are also observed in men, in different cultures/ethnicities or younger women remains unknown. Although women were initially recruited through the electoral roll, individuals who choose to participate in clinical trials tend to be healthier and more educated than the general population, which may introduce selection bias and limit generalisability. Nevertheless, despite women in this study presenting with higher socio-economic status, disease burden and pharmaceutical consumption were similar to data obtained from whole populations of this age.^11^ Another limitation is that the intervention period lasted five years, with the remaining follow-up being observational, and we did not capture calcium supplementation use beyond the trial period (after 2003). It is also important to acknowledge that the initial trial was not designed to evaluate cognition or dementia as either a primary or secondary outcome. Further, while education level is a known risk factor for dementia,^22^ this data was not available in our cohort. When a surrogate measure was considered instead (e.g., age at highest education level), our results remained comparable. We were also unable to assess cognitive function using a comprehensive battery of tests. However, we considered the AMS obtained at the end of the RCT and found no significant difference between the calcium and placebo groups. Although the AMS is highly specific, it has a known ceiling effect and may not capture subtle or early cognitive changes.^26^ Our study also considered all-cause dementia and did not ascertain whether there is an altered risk by dementia subtypes. Additionally, as the majority (90.7%) of dementia cases in our study occurred after the age of 80 years, a level of caution should be applied to interpreting the findings with respect to dementia events occurring before 80 years of age. Although our analysis considered total calcium intake (from both diet and supplements), it should be noted that mean dietary calcium intake in both the placebo and calcium groups were ∼960 mg/day, which is approximately 70% of generally recommended intakes for this age group (1300 mg).^27^ Hence, the generalisability of results to women with very low dietary calcium intakes may be limited and warrants further investigation. The use of linked hospital discharge administrative data has lower sensitivity (21.2%) but higher accuracy (96.7%) compared to chart review. While using such data increases the detection of dementia cases compared to using only death certificates, underreporting of dementia cases is a possibility.^16^ However, the 18.4% dementia incidence observed in this study over 14.5 years is comparable to other studies in similar populations (Australian women aged over 70 years, 20.4%) that used multiple sources of administrative data.^28^ Detecting dementia cases based on ICD-10 codes in hospital records has shown considerable agreement with chart reviews in other Australian studies,^29^ and continues to improve in sensitivity to detect dementia in hospital records up to ∼60% in more recent studies.^30^

In conclusion, this study provides evidence that five years of calcium carbonate supplementation (1200 mg per day) does not increase the long-term risk of developing dementia in community-dwelling older women. This finding is particularly important given the concerns raised in observational studies where the safety of calcium supplements has been highlighted when considering cognitive health.

## Contributors

NG, JRL, SRB, SML, BCMS, and MS: conceptualisation. RLP, KZ and JRL: investigation. NG, MS, and JRL: methodology and formal analyses. NG: writing-original draft. NG, JRL, SRB, SML, BCMS, JMH, KZ, RLP, and MS: writing-reviewing and editing. MS and NG had directly accessed and verified the underlying data reported and had primary responsibility for the final content. All authors read and approved the final manuscript.

## Data sharing statement

The data that support the findings of this study are available from the corresponding author upon reasonable request in-line with governing ethical considerations.

## Declaration of interests

We declare no competing interest.
