# Supplementary material for: Calcium supplementation and the risk of dementia in the Perth Longitudinal Study of Aging Women: a post-hoc analysis of a randomised clinical trial for fracture prevention
Source: Lancet Reg Health West Pac. 2025 Oct 6;63:101694. doi: 10.1016/j.lanwpc.2025.101694 (PMC12673806; doi:10.1016/j.lanwpc.2025.101694)
Supplement: Supplementary Tables [file mmc1.docx]

**Calcium supplementation and the risk of dementia in the Perth Longitudinal Study of Aging Women: a post-hoc analysis of a randomised clinical trial for fracture prevention.**

**Table of Contents**

**Supplementary Table 12**

**Supplementary Table 23**

**Supplementary Table 34**

**Supplementary Table 45**

**Supplementary Table 56**

**Supplementary Table 67**

**Supplementary Table 78**

**Supplementary Table 89**

**Supplementary Table 910**

**Supplementary Table 1011**

| **Supplementary Table 1: Baseline characteristics of participants by intervention groups.** | | |
| --- | --- | --- |
| **Baseline characteristics** | **Placebo** | **Calcium** |
| Number of participants (%) | 730 (50%) | 730 (50%) |
| ≥80% tablet adherence (per-protocol), n (%) | 410 (56·2%) | 420 (57·5%) |
| Age, years | 75·1± 2·7 | 75·2 ± 2·7 |
| Baseline dietary calcium intake ^1^, mg/day | 965 ± 354 | 956 ± 356 |
| Plasma 25OHD ^2^, nmol/L | 67·0 ± 29·6 | 67·0 ± 28·0 |
| Body mass index ^3^, kg/m^2^ | 27·4 ± 4·7 | 27·0 ± 4·8 |
| Ever smoked ^4^, n (%) | 259 (35·5%) | 280 (38·7%) |
| Systolic blood pressure ^5^, mmHg | 138·8 ± 18·8 | 137·0 ± 17·6 |
| Diabetes, n (%) | 47 (6·4%) | 48 (6·6%) |
| Statin medication, yes (%) | 147 (20·1%) | 129 (17·7%) |
| Low dose aspirin, yes (%) | 158 (21·6%) | 150 (20·5%) |
| Antihypertensive medication, yes (%) | 327 (44·8%) | 309 (42·3%) |
| Previous ASVD, n (%) | 99 (13·6%) | 79 (10·8%) |
| *APOE* genotypes ^6^ |  |  |
| *APOE* ε2/ε3, yes (%) | 97 (14·9%) | 107 (16·4%) |
| *APOE* ε2/ε4, yes (%) | 13 (2·0%) | 16 (2·5%) |
| *APOE* ε3/ε3, yes (%) | 412 (63·4%) | 381 (58·5%) |
| *APOE* ε3/ε4, yes (%) | 116 (17·8%) | 135 (20·7%) |
| *APOE* ε4/ε4, yes (%) | 12 (1·8%) | 12 (1·8%) |
| Physical activity ^7^, Kcal/day | 109 (0 – 196) | 113 (36 – 209) |
| Alcohol intake ^8^, g/day | 1·8 (0·3 – 10·0) | 1·8 (0·3 – 9·3) |
| Socio-economic status ^9^, n (%) |  |  |
| Top 10% most highly disadvantaged | 32 (4·4%) | 34 (4·7%) |
| Highly disadvantaged | 72 (9·9%) | 103 (14·2%) |
| Moderate – highly disadvantaged | 132 (18·2%) | 104 (14·3%) |
| Low - moderately disadvantaged | 108 (14·9%) | 116 (16·0%) |
| Low disadvantaged | 151 (20·9%) | 153 (21·1%) |
| Top 10% least disadvantaged | 229 (31·6%) | 215 (29·7%) |
| Data are expressed as mean ± SD, median (IQR), or n (%).  Abbreviations: 25-hydroxyvitamin D (25OHD), prevalent atherosclerotic vascular disease (ASVD), apolipoprotein E genotype (*APOE*), millimetres mercury (mmHg).  n= ^1^ 1445, ^2^ 1350, ^3^ 1458, ^4^ 1453, ^5^ 1412, ^6^ 1301, ^7^ 1458, ^8^ 1445, ^9^ 1449 | | |

| **Supplementary Table 2: Intention-to-treat hazard ratios for dementia events, hospitalisations, and deaths over 14·5 years, according to intervention groups.** | | | | |
| --- | --- | --- | --- | --- |
|  |  | **Placebo** | **Calcium** | **P value** |
|  | **Participants (n)** | 615 | 618 |  |
| ***Dementia events*** | **Events n (%)** | 118 (19·2) | 107 (17·3) |  |
|  | **Model 1** | Ref. | 0·89 (0·69, 1·16) | 0·41 |
|  | **Model 2** | Ref. | 0·86 (0·66, 1·11) | 0·26 |
|  | **Model 3** | Ref. | 0·87 (0·66, 1·13) | 0·31 |
| ***Dementia hospitalisations*** | **Events n (%)** | 105 (17.1) | 96 (15.5) |  |
|  | **Model 1** | Ref. | 0·90 (0·68, 1·19) | 0·47 |
|  | **Model 2** | Ref. | 0·85 (0·65, 1·13) | 0·28 |
|  | **Model 3** | Ref. | 0·88 (0·66, 1·17) | 0·39 |
| ***Dementia deaths*** | **Events n (%)** | 53 (8.6) | 41 (6.6) |  |
|  | **Model 1** | Ref. | 0·76 (0·51, 1·15) | 0·20 |
|  | **Model 2** | Ref. | 0·76 (0.50, 1·14) | 0·19 |
|  | **Model 3** | Ref. | 0·76 (0·50, 1·15) | 0·20 |
| Values are hazard ratios and 95% confidence intervals.  Model 1: Unadjusted.  Model 2: Age, body mass index, and apolipoprotein E genotype.  Model 3: Model 2 + systolic blood pressure, use of antihypertensive medication, previous diabetes, prescription of statin medications, use of low dose aspirin, prevalent atherosclerotic vascular disease, alcohol intake, smoking status, baseline dietary calcium intake, physical activity, and socio-economic status. | | | | |

| **Supplementary Table 3: Per-protocol hazard ratios for dementia events, hospitalisations, and deaths over 14·5 years, according to intervention groups.** | | | | |
| --- | --- | --- | --- | --- |
|  |  | **Placebo** | **Calcium** | **P value** |
|  | **Participants (n)** | 377 | 388 |  |
| ***Dementia events*** | **Events n (%)** | 67 (17·8) | 58 (14·9) |  |
|  | **Model 1** | Ref. | 0·84 (0·59, 1·19) | 0·33 |
|  | **Model 2** | Ref. | 0·82 (0·57, 1·17) | 0·27 |
|  | **Model 3** | Ref. | 0·84 (0·58, 1·21) | 0·35 |
| ***Dementia hospitalisations*** | **Events n (%)** | 57 (15.1) | 52 (13.4) |  |
|  | **Model 1** | Ref. | 0·88 (0·60, 1·29) | 0·53 |
|  | **Model 2** | Ref. | 0·85 (0·58, 1·24) | 0·41 |
|  | **Model 3** | Ref. | 0·89 (0·60, 1·31) | 0·55 |
| ***Dementia deaths*** | **Events n (%)** | 28 (7.4) | 23 (5.9) |  |
|  | **Model 1** | Ref. | 0·79 (0·45, 1·37) | 0·41 |
|  | **Model 2** | Ref. | 0·81 (0·46, 1·41) | 0·46 |
|  | **Model 3** | Ref. | 0·81 (0·45, 1·45) | 0·49 |
| Values are hazard ratios and 95% confidence intervals.  Model 1: Unadjusted.  Model 2: Age, body mass index, and apolipoprotein E genotype.  Model 3: Model 2 + systolic blood pressure, use of antihypertensive medication, previous diabetes, prescription of statin medications, use of low dose aspirin, prevalent atherosclerotic vascular disease, alcohol intake, smoking status, baseline dietary calcium intake, physical activity, and socio-economic status. | | | | |

| **Supplementary Table 4: Hazard ratios for dementia events, hospitalisations, and deaths over 14·5 years, according to intervention groups including the additional adjustment for age at highest education level.** | | | | |
| --- | --- | --- | --- | --- |
|  | **Intention-to-treat** |  |  |  |
|  |  | **Placebo** | **Calcium** | **P value** |
|  | **Participants (n)** | 609 | 615 |  |
| ***Dementia events*** | **Events n (%)** | 116 (19·0) | 105 (17·1) |  |
|  | **HR (95%CI)** | Ref. | 0·85 (0·65, 1·12) | 0·27 |
| ***Dementia hospitalisations*** | **Events n (%)** | 103 (16·9) | 94 (15·3) |  |
|  | **HR (95%CI)** | Ref. | 0·87 (0·65, 1·16) | 0·35 |
| ***Dementia Deaths*** | **Events n (%)** | 52 (8·5) | 40 (6·5) |  |
|  | **HR (95%CI)** | Ref. | 0·73 (0·47, 1·11) | 0·14 |
|  | **Per-protocol** |  |  |  |
|  |  | **Placebo** | **Calcium** | **P value** |
|  | **Participants (n)** | 373 | 386 |  |
| ***Dementia events*** | **Events n (%)** | 65 (17·4) | 57 (14·8) |  |
|  | **HR (95%CI)** | Ref. | 0·85 (0·58, 1·22) | 0·38 |
| ***Dementia hospitalisations*** | **Events n (%)** | 55 (14·7) | 51 (13·2) |  |
|  | **HR (95%CI)** | Ref. | 0·91 (0·61, 1·35) | 0·64 |
| ***Dementia Deaths*** | **Events n (%)** | 27 (7·2) | 23 (6·0) |  |
|  | **HR (95%CI)** | Ref. | 0·82 (0·45, 1·46) | 0·50 |
| Model adjusted for: age, body mass index, and apolipoprotein E genotype, systolic blood pressure, use of antihypertensive medication, previous diabetes, prescription of statin medications, use of low dose aspirin, prevalent atherosclerotic vascular disease, alcohol intake, smoking status, baseline dietary calcium intake, physical activity, socio-economic status, and age at highest education level. | | | | |

| **Supplementary Table 5: Competing risk (non-dementia death)* for dementia events over 14·5 years in the calcium supplemented compared to placebo group under both intention-to-treat and per-protocol criteria.** | | | | |
| --- | --- | --- | --- | --- |
|  |  | **Placebo** | **Calcium** | **P value** |
| ***Dementia events*** | ***Intention-to- treat*** |  | ***sHR (95%CI)*** |  |
|  | **Model 1** | Ref. | 0·91 (0·72, 1·16) | 0·46 |
|  | **Model 2** | Ref. | 0·86 (0·66, 1·11) | 0·26 |
|  | **Model 3** | Ref. | 0·86 (0·65, 1·13) | 0·30 |
|  | ***Per-protocol*** |  | ***sHR (95%CI)*** |  |
|  | **Model 1** | Ref. | 0·86 (0·61, 1·21) | 0·40 |
|  | **Model 2** | Ref. | 0·81 (0·57, 1·16) | 0·26 |
|  | **Model 3** | Ref. | 0·82 (0·56, 1·18) | 0·29 |
| * Fine and Gray’s proportional sub-hazards analyses.  Values are sub-distribution hazard ratios (sHR) and 95% confidence intervals.  Model 1: Unadjusted.  Model 2: Age, body mass index, and apolipoprotein E genotype.  Model 3: Model 2 + systolic blood pressure, use of antihypertensive medication, previous diabetes, prescription of statin medications, use of low dose aspirin, prevalent atherosclerotic vascular disease, alcohol intake, smoking status, baseline dietary calcium intake, physical activity, and socio-economic status. | | | | |

| **Supplementary Table 6: Age-adjusted hazard ratios for dementia events over 14·5 years, according to intervention groups in women with or without atherosclerotic vascular disease.** | | | | | |
| --- | --- | --- | --- | --- | --- |
|  |  |  | **Placebo** | **Calcium** | **P value** |
| ***Dementia events*** |  | **Participants (n)** | 99 | 79 |  |
|  | ***ASVD present*** | **Events n (%)** | 20 (20·2) | 19 (24·1) |  |
|  |  | **HR (95%CI)** | Ref. | 1·33 (0·70, 2·56) | 0·37 |
|  |  | **Participants (n)** | 631 | 651 |  |
|  | ***ASVD absent*** | **Events n (%)** | 121 (19·2) | 109 (16·7) |  |
|  |  | **HR (95%CI)** | Ref. | 0.85 (0·65, 1·10) | 0·22 |
| Values are hazard ratios (HR), and 95% confidence intervals (CI) adjusted for age at baseline.  Abbreviations: atherosclerotic vascular disease (ASVD). | | | | | |

| **Supplementary Table 7: Proportion of women with prevalent cerebrovascular accidents that did or did not experience a dementia-related event over follow-up.** | | | |
| --- | --- | --- | --- |
|  | **All participants** | **Placebo** | **Calcium** |
| ***Number of participants, n (%)*** | 55 | 34 (61·8) | 21 (38·2) |
| ***No dementia, n (%)*** | 41 (74·5) | 25 (73·5) | 16 (76·2) |
| ***Dementia, n (%)*** | 14 (25·5) | 9 (26·5) | 5 (23·8) |

| **Supplementary Table 8: Intention-to-treat hazard ratios for dementia events, hospitalisations, and deaths over 14·5 years, according to intervention groups including the additional adjustment for the presence of focal carotid plaques.** | | | | |
| --- | --- | --- | --- | --- |
|  |  | **Placebo** | **Calcium** | **P value** |
|  | **Participants (n)** | 511 | 518 |  |
| ***Dementia events*** | **Events n (%)** | 91 (17·8) | 78 (15·1) |  |
|  | **HR (95%CI)** | Ref. | 0·80 (0·59, 1·10) | 0·17 |
| ***Dementia hospitalisations*** | **Events n (%)** | 80 (15·7) | 68 (13·1) |  |
|  | **HR (95%CI)** | Ref. | 0·79 (0·57, 1·11) | 0·18 |
| ***Dementia deaths*** | **Events n (%)** | 41 (8·0) | 28 (5·4) |  |
|  | **HR (95%CI)** | Ref. | 0·68 (0·41, 1·13) | 0·14 |
| Values are hazard ratios (HR) and 95% confidence intervals (CI). Multivariable adjusted for age, body mass index, apolipoprotein E genotype, systolic blood pressure, use of antihypertensive medication, previous diabetes, prescription of statin medications, use of low dose aspirin, prevalent atherosclerotic vascular disease, alcohol intake, smoking status, baseline dietary calcium intake, physical activity, socio-economic status, and presence of focal carotid plaques. | | | | |

| **Supplementary Table 9: Intention-to-treat hazard ratios for dementia events, hospitalisations, and deaths over 14·5 years, according to intervention groups including the additional adjustment for carotid intimal-medial thickness (CIMT).** | | | | |
| --- | --- | --- | --- | --- |
|  |  | **Placebo** | **Calcium** | **P value** |
|  | **Participants (n)** | 505 | 508 |  |
| ***Dementia events*** | **Events n (%)** | 90 (17·8) | 73 (14·4) |  |
|  | **HR (95%CI)** | Ref. | 0·77 (0·56, 1·06) | 0·11 |
| ***Dementia hospitalisations*** | **Events n (%)** | 79 (15·6) | 65 (12·8) |  |
|  | **HR (95%CI)** | Ref. | 0·78 (0·56, 1·10) | 0·16 |
| ***Dementia deaths*** | **Events n (%)** | 41 (8·1) | 25 (4·9) |  |
|  | **HR (95%CI)** | Ref. | 0·62 (0·37, 1·04) | 0·07 |
| Values are hazard ratios (HR) and 95% confidence intervals (CI). Multivariable adjusted for age, body mass index, apolipoprotein E genotype, systolic blood pressure, use of antihypertensive medication, previous diabetes, prescription of statin medications, use of low dose aspirin, prevalent atherosclerotic vascular disease, alcohol intake, smoking status, baseline dietary calcium intake, physical activity, socio-economic status, and carotid intimal-medial thickness. | | | | |

| **Supplementary Table 10: Per-protocol incidence rates (per 1000 person-year) for dementia outcomes in women categorised into higher and lower total calcium intake, from both supplements and dietary sources.** | | |
| --- | --- | --- |
| **Total calcium intake** | **<1610* mg/day** | **≥1610* mg/day** |
| **Participants (n)** | 413 | 413 |
| ***Dementia events*** | 12·63 | 12·73 |
| ***Dementia hospitalisations*** | 11·10 | 11·16 |
| ***Dementia deaths*** | 4·87 | 5·14 |
| *Median total calcium intake in the per-protocol subgroup. | | |
